# Patient-specific arterial input function for accurate perfusion assessment in intraoperative fluorescence imaging

**DOI:** 10.1117/1.JBO.29.S3.S33306

**Published:** 2024-09-06

**Authors:** Yue Tang, Shudong Jiang, Joseph S. Sottosanti, Thomas Usherwood, Xu Cao, Logan M. Bateman, Lillian A. Fisher, Eric R. Henderson, Ida Leah Gitajn, Jonathan Thomas Elliott

**Affiliations:** a Thayer School of Engineering at Dartmouth, Hanover, New Hampshire, United States; b Dartmouth Health, Department of Orthopaedics, Lebanon, New Hampshire, United States

**Keywords:** arterial input function, pulse dye densitometry, dynamic contrast-enhanced fluorescence imaging, indocyanine green, vascular perfusion, kinetic parameters of perfusion

## Abstract

**Significance:**

The arterial input function (AIF) plays a crucial role in correcting the time-dependent concentration of the contrast agent within the arterial system, accounting for variations in agent injection parameters (speed, timing, etc.) across patients. Understanding the significance of the AIF can enhance the accuracy of tissue vascular perfusion assessment through indocyanine green–based dynamic contrast-enhanced fluorescence imaging (DCE-FI).

**Aim:**

We evaluate the impact of the AIF on perfusion assessment through DCE-FI.

**Approach:**

A total of 144 AIFs were acquired from 110 patients using a pulse dye densitometer. Simulation and patient intraoperative imaging were conducted to validate the significance of AIF for perfusion assessment based on kinetic parameters extracted from fluorescence images before and after AIF correction. The kinetic model accuracy was evaluated by assessing the variability of kinetic parameters using individual AIF versus population-based AIF.

**Results:**

Individual AIF can reduce the variability in kinetic parameters, and population-based AIF can potentially replace individual AIF for estimating wash-out rate ($k_{\mathrm{ep}}$), maximum intensity ($I_{\max}$), ingress slope with lower differences compared with those in estimating blood flow, volume transfer constant ($K^{\mathrm{trans}}$), and time to peak.

**Conclusions:**

Individual AIF can provide the most accurate perfusion assessment compared with assessment without AIF or based on population-based AIF correction.

## 1 Introduction

Perfusion plays an important role in bone health. Adequate bone perfusion is critical to supporting fracture healing and preventing infection.^1^ Because of this, thorough debridement of poorly perfused bone is fundamental for treating severe open, contaminated fractures.^2^ In the absence of intraoperative methods to measure bone perfusion accurately, the extent of debridement is subjective and depends on a surgeon’s experience, which may contribute to the high variability in outcomes, especially with respect to surgical site infection.^3^ To address this, we have advanced an indocyanine green (ICG)-based dynamic contrast-enhanced fluorescence imaging (DCE-FI) technique. This innovative approach enables real-time measurement of bone perfusion during surgery. Termed “fluorescence-guided debridement,” this technique has the potential to enhance surgical precision by guiding the resection of devitalized bone.^4^

Fluorescence-guided debridement depends on the quantitative assessment of tissue perfusion, through the measurement of fluorescence intensity during the wash-in and wash-out of dye. These measurements are then processed to recover either standardized images of fluorescence intensity or ICG concentration or parametric maps of hemodynamic (also called kinetic) parameters. Accurate recovery of quantitative maps involves measuring both the tissue concentration over time and the arterial concentration in the arteries that deliver the dye to the tissue of interest. This time-dependent concentration of contrast agent in the arterial system is called the arterial input function (AIF). As we have previously shown,^5^ AIF measurement is critical for the proper interpretation of DCE-FI because AIF is influenced by a number of modifiable and non-modifiable factors. For example, in the operating room, the contrast agent is administered to patients manually (without the use of a syringe pump) by anesthesiologists. Despite training on best practices for administering this bolus injection, we observed high variability in injection parameters such as injection speed, timing, and peak dose; these features undermine the accuracy of perfusion assessment by DCE-FI.^5^ Also, the acquisition of the AIF is essential for quantifying perfusion-related parameters in tracer kinetic modeling.^6^ Given the convolution theory of tracer kinetics,^7^ tissue concentration of the contrast agent is approximated by convolving AIF with an impulse residue function, where parameters such as blood flow are extracted. This modeling process cannot be achieved without a valid AIF.^8^^,^^9^

Various methods have been utilized to measure AIF. Although direct blood sampling for measuring ICG concentration is considered the gold standard, it is invasive and impractical for routine clinical use.^10^ Alternatively, as a non-invasive method, image-derived AIF can be estimated from the regions of interest (ROIs) placed at the location of the arteries during the imaging sessions.^11^ Although this method does not require additional hardware, it is limited by the imaging’s temporal resolution, susceptibility to noise, dependency on the accuracy of identifying arterial structures, and contamination from adjacent and underlying pixels.^12^ Thus, traditional imaging modalities often resort to using population-based average AIF (${\mathrm{AIF}}_{\mathrm{POP}}$) instead of patient-specific individual AIF (${\mathrm{AIF}}_{\mathrm{IND}}$).^13^ Although ${\mathrm{AIF}}_{\mathrm{POP}}$ does not account for patient variations, studies have shown no significant differences in kinetic parameters extracted based on either ${\mathrm{AIF}}_{\mathrm{POP}}$ or ${\mathrm{AIF}}_{\mathrm{IND}}$,^14^^–^^19^ with some reporting reduced variability when using ${\mathrm{AIF}}_{\mathrm{POP}}$.^13^^,^^20^

In contrast to the above methods to obtain AIF, a non-invasive pulse dye densitometer (PDD) has also been developed and deployed in blood flow measurements, primarily in the context of absorption-based near-infrared spectroscopy (NIRS) tissue probes.^21^ PDD is based on the ratio of arterial pulsatile absorbance signals at two wavelengths measured through the finger clip of an oximeter.^22^ Compared with other AIF measurements, PDD can have high temporal resolution and avoid the noise and artifacts caused by inaccuracies of ROI size and location selection of the arterial blood vessel.^23^ Initially, commercial PDD devices were developed for liver function tests^24^ and cardiac output evaluations;^25^^,^^26^ however, these are not widely available in clinical practices. Studies deploying PDD in combination with NIRS detection of ICG dynamics have been demonstrated in neonates^27^ and adults^28^ in the neuro-intensive care unit.

In our prior work, we have demonstrated the significant role of AIF in correcting perfusion assessment in DCE-FI with ${\mathrm{AIF}}_{\mathrm{IND}}$ through simulation studies.^5^ In this study, to validate the importance of AIF for perfusion assessment using DCE-FI, we utilized PDD to acquire real AIFs from patients who underwent orthopedic surgeries, examined the variations in AIFs across patients, and assessed their impact on modeled kinetic parameters. This was achieved by comparing the variability of kinetic parameters directly extracted from fluorescence images before and after AIF correction. In addition, to evaluate the different methods of AIF correction, both ${\mathrm{AIF}}_{\mathrm{IND}}$ and ${\mathrm{AIF}}_{\mathrm{POP}}$ were utilized through kinetic modeling, and perfusion-related parameters derived from these two types of AIFs were compared.

## 2 Materials and Methods

### 2.1 Instrumentation and Data Acquisition

**Fig. 1:**
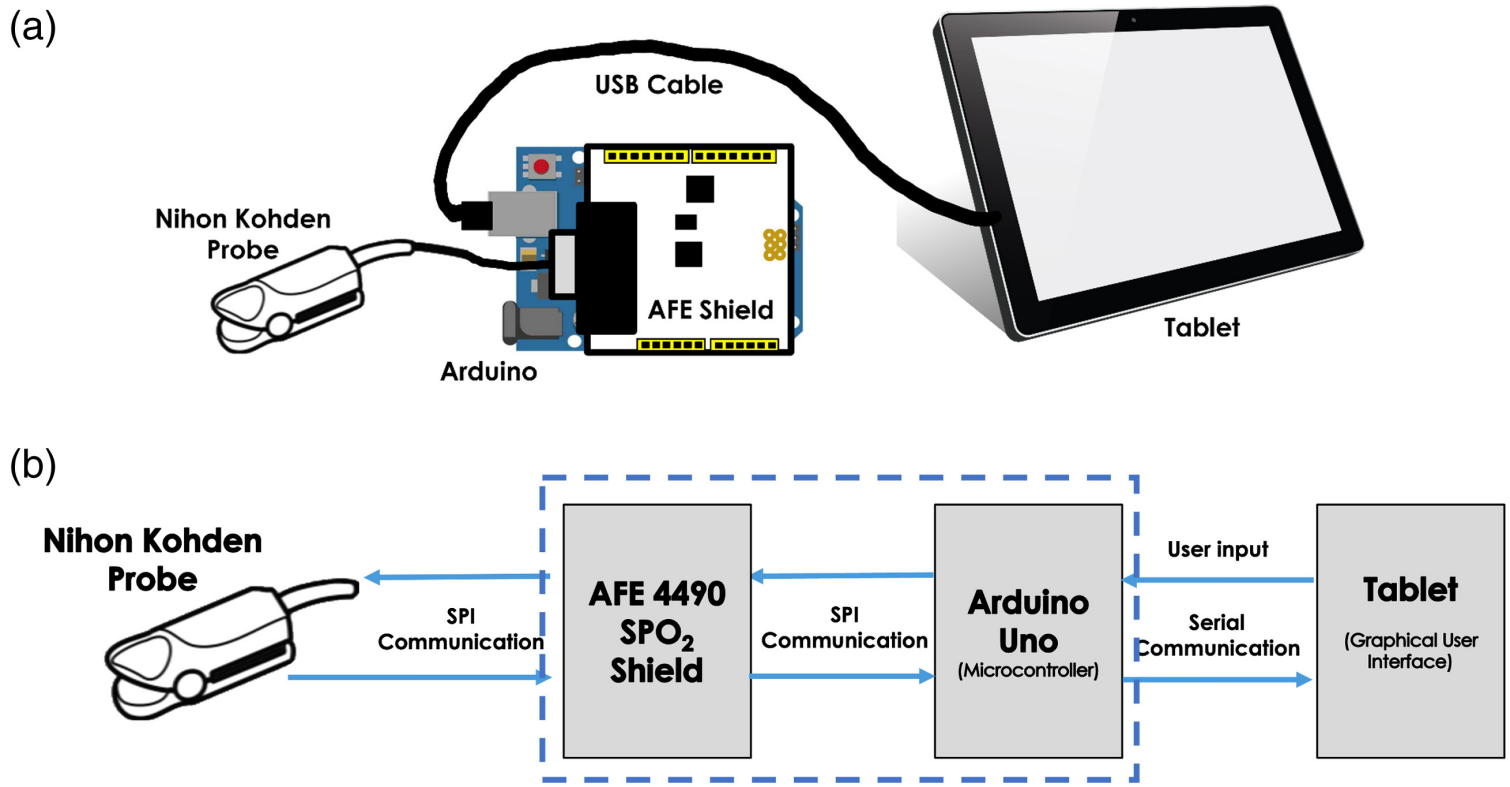
(a) Device setup and (b) schematic diagram of a pulse dye densitometer based on an oximeter finger probe.

Figure 1 illustrates the diagram of the AIF data collection. AIFs were obtained by an in-house-developed PDD based on a commercial pulse oximeter device (AFE4490, Texas Instruments, Dallas, Texas, United States). The existing system consisted of an integrated analog front-end circuit board and a standard two-wavelength pulse oximetry probe (660 and 940 nm).^5^ The modifications included replacing a standard oximeter finger probe with an ICG-sensitive finger probe that uses 805 and 940 nm light-emitting diodes (LEDs; TL-301P, Nihon Kohden, Tokyo, Japan), along with a custom-developed data processing software installed on a tablet [Fig. 1(a)]. As the absorption of ICG is at its maximum at 805 nm while it is nearly zero at 940 nm, by detecting and calculating the fractional pulsatile signal change of the diffused light (through the finger tissue) at each of these two wavelengths, the concentrations of ICG at certain time point can be obtained.^22^ As shown in Fig. 1(b), AFE4490 Pulse Oximeter Shield contains a pulse oximetry integrated circuit that allows control and digitization of detected optical signals acquired at a sampling rate of 300 Hz. As AIF was extracted from the amplitudes of pulsed signals in each cardiac cycle, the sampling rate of AIF depends on the heart rate of patients, ranging from 0.8 to 1.5 Hz. An in-house-developed code in the tablet communicates with the Arduino via a Serial Peripheral Interface to collect data from the pulse oximeter probe via the AFE Shield. AIF data acquisition started 2 min before and ended simultaneously with DCE-FI.

Fluorescence imaging was conducted using a SPY Elite Fluorescence imaging system (Stryker, Kalamazoo, Michigan, United States). In this system, near-infrared light at the wavelength of 805 nm was utilized to stimulate the intravenously administered ICG. A charge-coupled device camera equipped with an 820- to 900-nm bandpass filter was utilized for capturing fluorescence imaging, and an RGB camera was connected through a beam splitter for white-light image acquisition. The system was positioned 30 cm away from the field of view (FOV). Each imaging session involved acquiring fluorescence videos every 3.75 frames per second for a total of 1024 frames. Further details regarding the system specifications can be found in our previous publications.^29^

### 2.2 Patient Study

This study was conducted in accordance with the Health Insurance Portability and Accountability Act and received approval by the institutional review board at Dartmouth Health, Lebanon, New Hampshire, United States. Written informed consents were obtained from 110 patients who underwent open orthopedic surgeries. Within these 110 patients, 15 patients who underwent lower extremity amputation had three individual imaging sessions as follows: (1) baseline, representing bone without damage; (2) osteotomy at the tibial diaphysis, representing a simple fracture; and (3) circumferential periosteal/soft tissue stripping of the entire tibia, modeling extremely severe injury with extensive periosteal degloving. For the other patients with either infections or open fractures, one or two imaging sessions were carried out after debridement.

During each imaging session, we acquired AIF and ICG-based DCE-FI simultaneously. After 20 s of pre-injection imaging, 0.1  mg/kg of ICG was intravenously administered to the patient. The fluorescence imaging automatically ended after the complete acquisition of 1024 frames for a duration of ∼4.5  min. AIF collection was manually terminated after the imaging was accomplished. In total, 144 high-quality AIFs have been obtained. These AIFs have clear, plausible shapes based on ICG recirculation in the arterial system and exhibit very limited noise. Specifically, these AIFs show a rapid initial increase, a gradual wash-out phase without decreasing below 0, and a smooth curve without irregular fluctuations.

### 2.3 AIF Data Analysis

**Fig. 2:**
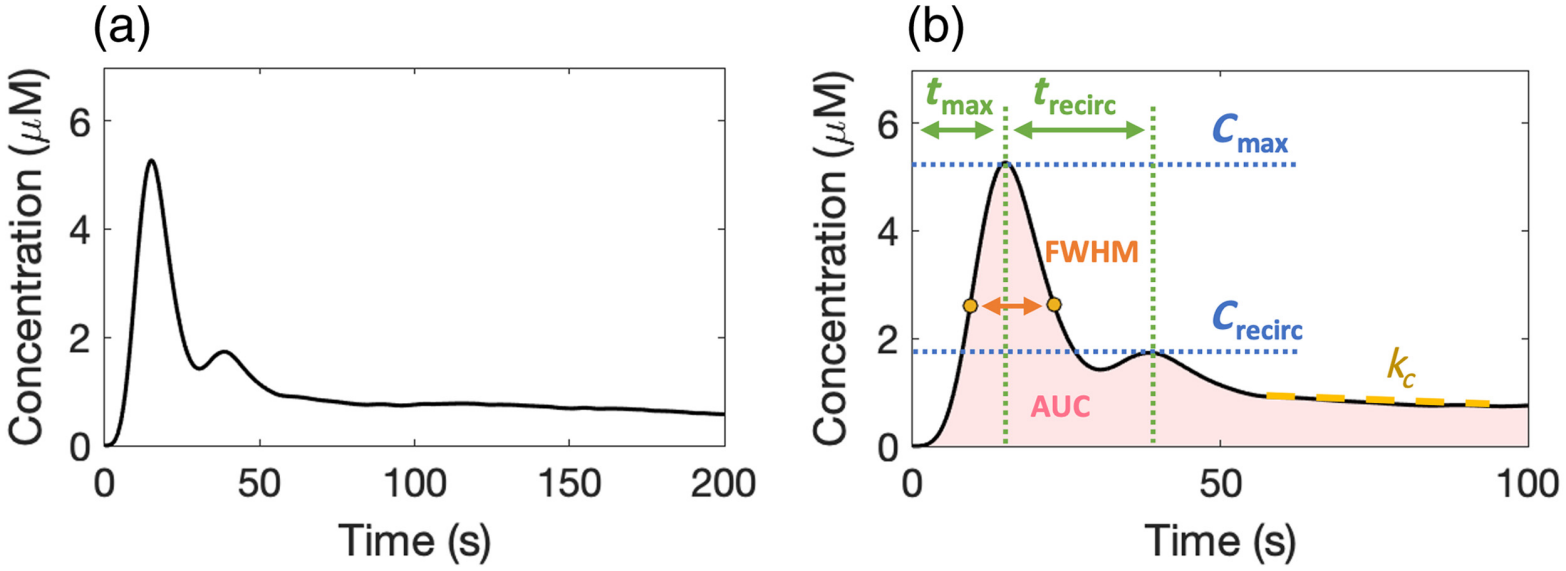
(a) Example of arterial input function describing ICG concentration versus time, annotated with (b) relevant kinetic parameters extracted from the AIF curve.

Figure 2(a) demonstrates a typical example of AIF collected by the pulse oximeter–based PDD, featuring a prominent first-pass peak and a delayed second peak attributed to ICG recirculation. After perfusing into the tissue, ICG returns to the venous system, transports to the heart, and re-enters the arterial system. It may also recirculate through other organs such as kidneys and lymph nodes.^6^ The recirculation peak is lower and broader due to dispersion and mixing, with a time delay between the first and the recirculated peak. Figure 2(b) illustrates relevant AIF parameters extracted from the curve, including maximum concentration ($C_{\max}$), time to maximum concentration ($t_{\max}$), the concentration of the recirculation peak ($C_{\text{recirc}}$), the time of recirculation delay ($t_{\text{recirc}}$), full width at half maximum of the first-pass peak, the area under the curve, and the clearance rate ($k_{c}$).

To present the variations in AIF curves, 144 ${\mathrm{AIF}}_{\mathrm{IND}}$ from 110 patients were displayed case by case on the same scale for comparison. Each subfigure displayed one to three AIFs collected from the same case while maintaining the same range on the X and Y axes. The means and standard deviations of AIF parameters such as $C_{\max}$ and $t_{\max}$ were calculated.

Pearson correlation coefficients were calculated between each patient variable and each AIF parameter to assess whether variations in AIFs can be explained by patient information. For visualization purposes, the correlation coefficients were displayed in heatmaps, with positive and negative correlation coefficients colored in red and blue, respectively. The near-zero correlation coefficient was represented in white.

As averaging AIF from the entire cohort (N=144) would smooth out the characteristic recirculation peaks, potentially increasing errors in kinetic analysis, ${\mathrm{AIF}}_{\mathrm{POP}}$ was averaged from 60 ${\mathrm{AIF}}_{\mathrm{IND}}$ with minimal noise and typical AIF features, including a recirculation peak with its prominence higher or equal to 5% of the first peak ($C_{\max}$). The selected ${\mathrm{AIF}}_{\mathrm{IND}}$ were interpolated to match the sampling rate of fluorescence imaging (3.75 frames per second), with the arrival time adjusted to the origin. Subsequently, they were averaged at each time point, with standard deviations (SDs) calculated.

### 2.4 Simple Curve and Adiabatic Approximation to the Tissue Homogeneity (AATH) Modeling Analysis

To assess the tissue and bone blood perfusion, perfusion-related kinetic parameters based on the ICG time–intensity curve or the AATH modeling were analyzed.^30^

**Fig. 3:**
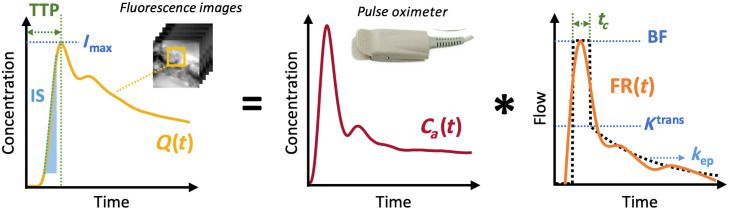
Parameters of simple time–concentration curve and AATH model analysis, for assessing bone perfusion using ICG-based DCE-FI and AIF. The ICG concentration in the tissue Q(t) is the convolution of the ICG concentration in the arterial system $C_{a}(t)$ and the flow-scaled impulse response function FR(t).

As shown in Fig. 3, based on the ICG time–intensity curve, the perfusion can be represented by simple kinetic parameters, such as maximum fluorescence intensity ($I_{\max}$), time to peak (TTP), and ingress slope (IS).^31^ For fairly comparing these parameters among cases, tissue ICG concentration Q(t) was normalized by first deconvolving by its patient-specific individual AIF ($C_{a}(t))$ and then re-convolving with $C_{a}^{\prime }(t)$, a standard AIF (${\mathrm{AIF}}_{\mathrm{STD}}$) selected from a high-quality AIF of a patient case, as follows:

$$Q^{\prime }(t)=C_{a}^{\prime }(t)*\text{deconv}(Q(t),C_{a}(t)).$$ (1)

As the same $C_{a}^{\prime }(t)$ was applied for each case, the differences of the patient-specific individual AIF $C_{a}(t)$ caused by the manual injection were eliminated. The $C_{a}^{\prime }(t)$ that we applied in the study has a peak concentration near the mean peak value of all 144 datasets, a clear plausible shape based on ICG recirculation in the arterial system, and very limited noise. Although choosing a different $C_{a}^{\prime }(t)$ may influence the absolute values of $Q^{\prime }(t)$, the differences in simple kinetic parameters among cases will not be affected.

The deconvolution was achieved by least square approximation.^32^ A regularization parameter λ was added into the solution of ordinary least squares to ensure the invertibility of the matrix, as follows:

$$X=(H^{T}H+\lambda I{)}^{-1}H^{T}Q(t),$$ (2)

where H is the Toeplitz matrix of $C_{a}(t)$, and I is the identity matrix. A value of $\lambda =10^{-3}$ was tested to adequately attenuate noise while maintaining data accuracy across the patient datasets and was applied in the deconvolution process.

In addition to the simple kinetic analysis performed on the dynamic ICG fluorescence curves to quantify perfusion, the AATH model was also applied, which accounts for the time-dependent hemodynamics attributed to the structure of tissue/bone blood supply.^8^^,^^30^ The approach is based on the convolution theory of tracer kinetics and is summarized graphically in Fig. 3, where the * sign represents the convolution operator. The relationship among the concentrations of ICG in tissue and arterial blood can be given by the following equation:^5^

$$Q(t)=C_{a}(t)*\mathrm{FR}(t),$$ (3)

where Q(t) is the time–concentration curve of ICG in the tissue, derived from the average intensity within a designated ROI of motion-corrected fluorescence images. $C_{a}(t)$ is the concentration curve of ICG in the arterial blood. FR(t) is the flow-scaled impulse residue function [FR(t)=BF·R(t), where BF is the blood flow and R(t) is the ratio of remaining ICG at time t following an idealized bolus (a delta function)].

To solve Eq. (3), the AATH model was applied to approximate R(t) into two phases, vascular $R_{v}(t)$ and parenchymal $R_{p}(t)$, which can be defined as

$$R_{v}(t)=1-\Theta (t-t_{c}),$$ (4)

$$R_{p}(t)=Ee^{k_{\mathrm{ep}}(t-t_{c})}\Theta (t-t_{c}),$$ (5)

$$Q(t)=C_{a}(t)*\mathrm{BF}[R_{v}(t)+R_{p}(t)],$$ (6)

where E is the extraction fraction, $t_{c}$ is the capillary transit time, $k_{\mathrm{ep}}$ is the rate transfer constant between extracellular extravascular space and intravascular space, and $\Theta (t-t_{c})$ is the Heaviside function with transition at $t_{c}$. Other relevant kinetic parameters such as volume transfer constant ($K^{\mathrm{trans}}$) can be derived as $K^{\mathrm{trans}}=E\cdot \mathrm{BF}$.

### 2.5 Simulation Study for Evaluating the Accuracy of Kinetic Parameters in the Perfusion Assessment by ICG-Based DCE-FI

In our prior simulation study,^5^ simulated AIFs were utilized to highlight the significance of AIF in perfusion assessment. In this study, 144 patient AIFs were employed to evaluate the accuracy of the relevant kinetic parameters in the perfusion assessment based on ICG-based DCE-FI.

The ICG time–concentration curves from DCE-FI were categorized into three typical intensity enhancement curves as follows: type I (persistent)—a constantly increasing signal intensity without clear wash-out; type II (plateau)—an initial peak followed by a relatively constant signal; and type III (wash-out)—a sharp peak followed by a substantially decreasing intensity.^9^

To simulate these three representative curves, three original impulse residue functions, denoted as $R_{0}(t)$, were extracted from real impulse residue functions based on the different levels of $k_{\mathrm{ep}}$ ($k_{\mathrm{ep}}=0.03$, 0.16, and $0.50\;\min^{-1}$, respectively). $R_{0}(t)$ was then convolved with ${\mathrm{AIF}}_{\mathrm{POP}}$ or patient-specific ${\mathrm{AIF}}_{\mathrm{IND}}$ to generate the simulated time–concentration curves. By fitting the simulated time–concentration curves to the AATH model, the modeled impulse residue functions, $R_{1}(t)$, were extracted. The accuracy of each kinetic parameter was evaluated by calculating the error ratio $(R_{1}(t)-R_{0}(t))/R_{0}(t)\cdot 100\%$ for each enhancement type.

For perfusion-related parameters in simple curve analysis, the simulated time–concentration curves were deconvolved by either individual or population-based AIFs and then re-convolved with the same AIF, with the error ratios calculated for each parameter.

### 2.6 Kinetic Analysis

For skin tissue, we analyzed the kinetic parameters within the intact skin area of a patient who underwent two imaging sessions with a 20-min time separation, assuming the blood perfusion in the normal intact skin area is consistent over two consecutive imaging sessions. Fifteen circular ROIs with a radius of 4 mm were selected on the skin regions located at least 2 cm away from the incision. Kinetic parameters, including $I_{\max}$, TTP, IS, BF, $K^{\mathrm{trans}}$, and $k_{\mathrm{ep}}$, were extracted from these ROIs based on ${\mathrm{AIF}}_{\mathrm{IND}}$ and ${\mathrm{AIF}}_{\mathrm{POP}}$. In addition, $I_{\max}$, TTP, and IS before any AIF correction were derived from the average fluorescence intensity curve of each ROI from the original imaging data, which were compared with those results after AIF correction.

Paired-sample t-tests were conducted to determine whether these parameters significantly differed between the two imaging sessions. When we combined each parameter extracted from both imaging sessions, the variability of each parameter was assessed using the coefficient of variation (CoV), calculated with the following equation:

$$\mathrm{CoV}=\frac{\sigma }{\mu }\cdot 100\%,$$ (7)

where μ and σ represent the mean and standard deviation of the population, respectively. A smaller CoV indicates better consistency over two imaging sessions.

For bone tissue analysis, kinetic parameters were computed using both ${\mathrm{AIF}}_{\mathrm{IND}}$ and ${\mathrm{AIF}}_{\mathrm{POP}}$ within the bone regions from 144 imaging sessions. Surgeons manually delineated exposed bone regions with the assistance of white light and fluorescence imaging. Within these delineated regions, the average values of each parameter were derived. The average values obtained from ${\mathrm{AIF}}_{\mathrm{IND}}$ were compared with those obtained through ${\mathrm{AIF}}_{\mathrm{POP}}$ through a linear regression. Key variables such as slope, intercept, coefficient of determination ($R^{2}$), and p-value of the paired t-test were calculated to assess the relationships and disparities of each parameter corrected by ${\mathrm{AIF}}_{\mathrm{IND}}$ and ${\mathrm{AIF}}_{\mathrm{POP}}$.

The data were also presented in Bland–Altman plots, with the X and Y axes representing the means and the differences of the average values of each parameter extracted based on ${\mathrm{AIF}}_{\mathrm{POP}}$ and ${\mathrm{AIF}}_{\mathrm{IND}}$ in each case, respectively. The differences were computed by subtracting the parameters based on ${\mathrm{AIF}}_{\mathrm{IND}}$ from those based on ${\mathrm{AIF}}_{\mathrm{POP}}$ (${\mathrm{AIF}}_{\mathrm{POP}}-{\mathrm{AIF}}_{\mathrm{IND}}$). Relevant variables were also derived to analyze the range and significance of differences for each parameter, including the mean difference, SD, and limits of agreement (LOA), defined as the mean difference ±1.96 SD.^33^

To analyze the differences at various perfusion levels in the bone tissues, parameter differences were assessed specifically in the amputation cohort with 11 patients that have high-quality AIFs for each of the three imaging sessions. Each imaging session exhibited a significantly different level of perfusions, from the highest (baseline) to intermediate (osteotomy) to the lowest (soft tissue stripping). Similar to our previous study focused on the amputation cohort,^29^ the kinetic parameters were extracted from four representative ROIs (with a 10-mm diameter positioned on the tibia with an equal distance along the central axis). In each imaging session, $R^{2}$ of each kinetic parameter was derived from linear regression between the results based on ${\mathrm{AIF}}_{\mathrm{POP}}$ and ${\mathrm{AIF}}_{\mathrm{IND}}$.

## 3 Results

### 3.1 Patient AIFs and Correlations with Clinical Parameters

The patient information, including weight, height, body mass index (BMI), age, hemoglobin, and sex of 110 patients involved in this study, is summarized in Table 1. There are no significant differences in weight, BMI, and hemoglobin among the cohorts. However, the ages of patients with open fractures are notably lower than those in the infection and amputation cohorts, whereas the heights of patients with infection are significantly lower than those of patients with amputation (p<0.05).

**Table 1:** Summary of patient information (N=110).

	Open fracture (n=42)	Infection (n=53)	Amputation (n=15)	All patients (n=110)
Weight (kg) [SD]	93.8 [26.9]	93.4 [32.1]	101.7 [35.9]	94.7 [30.6]
Height (cm) [SD]	174.8 [8.5]	172.0 [9.2]	177.5 [9.0]	173.9 [9.0]
BMI ($\mathrm{kg}/m^{2}$) [SD]	30.7 [8.1]	31.2 [11.0]	31.7 [10.1]	31.1 [9.8]
Age (years) [SD]	43.5 [16.6]	52.8 [15.5]	55.6 [15.1]	49.6 [16.5]
Hemoglobin (g/dL) [SD]	11.4 [2.7]	11.5 [2.3]	10.4 [1.9]	11.3 [2.4]
Biological sex (M/F)	30/12	28/25	13/2	71/39

**Fig. 4:**
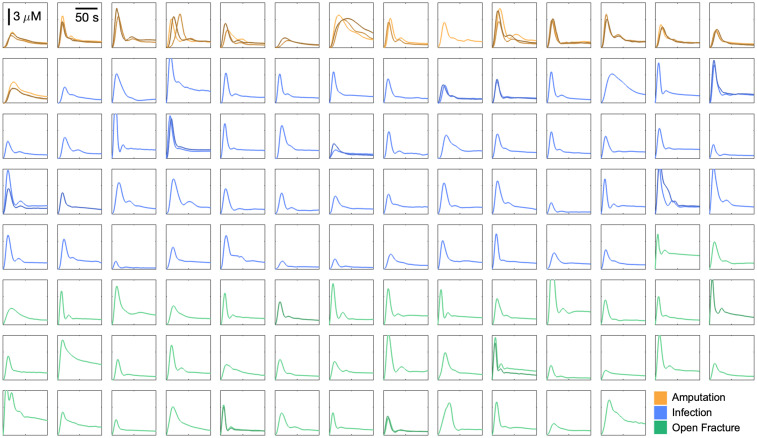
A total of 144 arterial input functions from 110 patients across three patient cohorts. The Y-axis is the concentration in micromolars, and the X-axis is the time in seconds. All traces are displayed with the same scale, shown by scale bars in the top left corner. For panels with more than one trace, each serial ICG injection is displayed with an increasingly darker shade.

Figure 4 shows the individual AIFs obtained from 144 intraoperative imaging sessions. Each subfigure displays one to three AIFs collected from the same case while maintaining the same range on the X (0 to 100 s) and Y (0 to 8  μM) axes. AIFs from the patient cohort of amputation, infection, and open fracture are colored in yellow, blue, and green, respectively. For cases with more than one imaging session, the AIFs are colored in darker shades with the increasing imaging number. Maximum concentration $C_{\max}$ varies from 1.3 to 12.7  μM (mean, 4.9  μM; SD, 2.0  μM), whereas time to maximum concentration $t_{\max}$ ranges from 5.9 to 42.1 s (mean, 13.3 s; SD, 5.3 s).

**Fig. 5:**
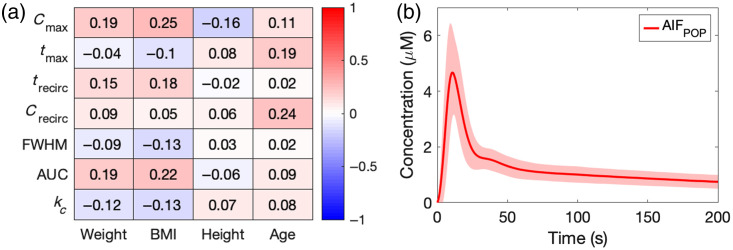
(a) Correlation coefficient heatmap showing the relationship between AIF parameters and patient clinical variables. Positive coefficients are colored in red, whereas negative coefficients are colored in blue. (b) Population-based AIF (${\mathrm{AIF}}_{\mathrm{POP}}$) averaged from 60 high-quality AIFs with standard deviations shaded in red.

Figure 5(a) presents the correlation coefficient heatmap between AIF parameters and patient variables, with positive and negative values colored in red and blue, respectively. The absolute values of correlation coefficients are varied between 0.02 and 0.25, indicating that none of the AIF parameters correlate to any clinical parameters significantly. Figure 5(b) shows the ${\mathrm{AIF}}_{\mathrm{POP}}$ averaged from 60 high-quality ${\mathrm{AIF}}_{\mathrm{IND}}$ with minimal noise and typical AIF features, including recirculation peaks, whereas its SDs were shaded in red.

### 3.2 Tracer Kinetic Modeling Error Analysis

**Fig. 6:**
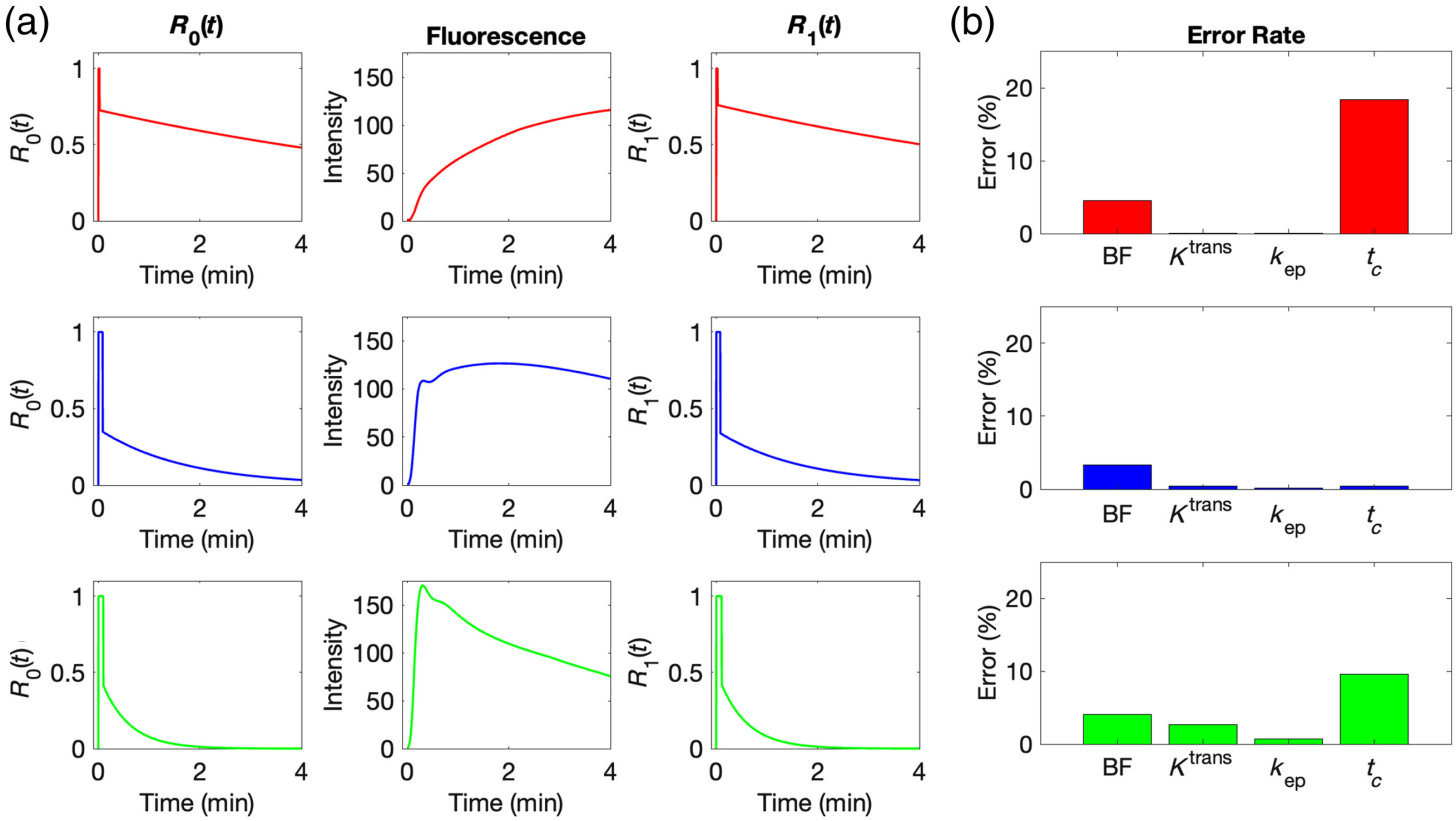
Simulation (a) diagram and (b) errors when using ${\mathrm{AIF}}_{\mathrm{POP}}$ in AATH model fitting. Rows, from top to bottom, show the type I (persistent), type II (plateau), and type III (wash-out) patterns. Columns, from left to right, feature the original impulse residue function $R_{0}(t)$, representative fluorescence curve by convolving $R_{0}(t)$ with ${\mathrm{AIF}}_{\mathrm{POP}}$ [unit: relative fluorescence units (RFUs)], modeled impulse residue function $R_{1}(t)$, and error rates between $R_{0}(t)$ and $R_{1}(t)$.

Figure 6 shows the simulation result for ${\mathrm{AIF}}_{\mathrm{POP}}$ based on three typical intensity enhancement curves. As shown in Fig. 6(a), $R_{0}(t)$ (left column) were three original impulse residue function curves based on real ICG concentration curves with $k_{\mathrm{ep}}$ of $0.03\;\min^{-1}$ (top row), $0.16\;\min^{-1}$ (middle row), and $0.50\;\min^{-1}$ (bottom row). $R_{0}(t)$ were then convolved with ${\mathrm{AIF}}_{\mathrm{POP}}$ to generate representative time–concentration curves corresponding to those three typical time–concentration curves (middle column). The same AIF was then applied to the AATH modeling, and the corresponding modeled impulse residue function $R_{1}(t)$ was derived (right column). Finally, $R_{1}(t)$ was compared with its corresponding $R_{0}(t)$, and the errors in each kinetic parameter were calculated. The results in Fig. 6(b) demonstrate that besides $t_{c}$, the errors of each of BF, $K^{\mathrm{trans}}$, and $k_{\mathrm{ep}}$ were all below 5%, indicating a satisfactory fit in the modeling results.

When using ${\mathrm{AIF}}_{\mathrm{POP}}$ and the simulated three typical time–concentration curves for simple kinetic analysis, the errors of each kinetic parameter, including maximum intensity ($I_{\max}$), time to peak (TTP), and ingress slope (IS), due to the deconvolution and re-convolution process were all less than 1%.

**Fig. 7:**
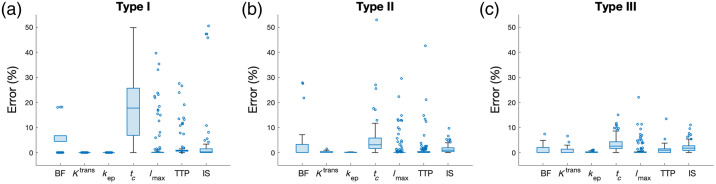
Simulation results for errors of each kinetic parameter when using each of 144 ${\mathrm{AIF}}_{\mathrm{IND}}$ in (a) type I, (b) type II, and (c) type III ICG time–concentration curves shown in Fig. 6.

Instead of ${\mathrm{AIF}}_{\mathrm{POP}}$, Fig. 7 presents the statistical results of errors when utilizing 144 real ${\mathrm{AIF}}_{\mathrm{IND}}$ in each of the simulated three typical time–concentration curves. BF, $K^{\mathrm{trans}}$, $k_{\mathrm{ep}}$, $I_{\max}$, TTP, and IS consistently demonstrate low percentage errors across all three typical types, which implies that these parameters are less sensitive to the changes in time–concentration types, making them suitable for perfusion assessments by DCE-FI. Similar to the results when using ${\mathrm{AIF}}_{\mathrm{POP}}$, the errors in the parameter of $t_{c}$ are notably higher, especially in type I, suggesting potential compromises in the accuracy of extracting $t_{c}$.

### 3.3 Evaluation of Variation in Patient Skin

**Fig. 8:**
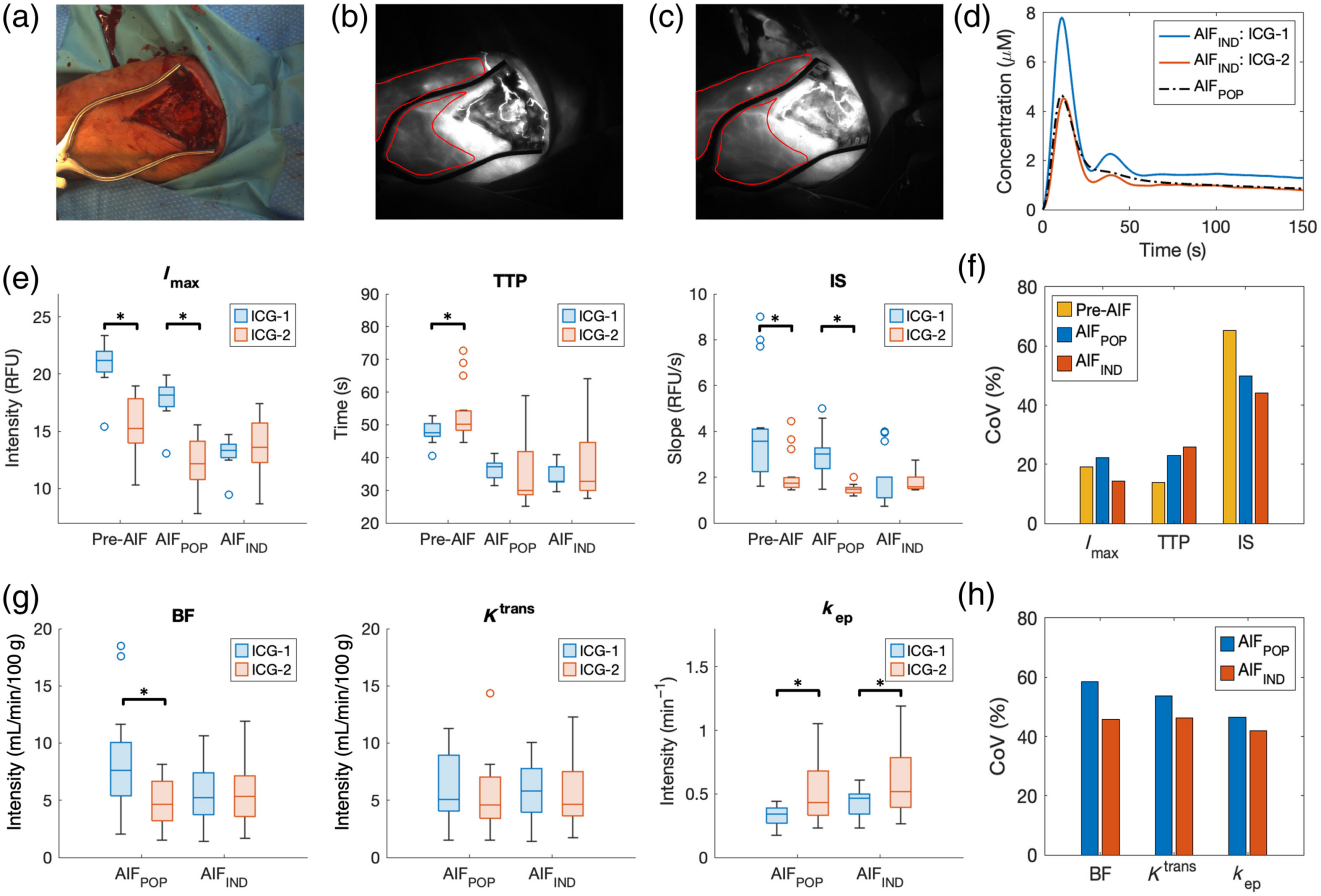
Comparison of the kinetic parameters in the skin areas of a case involving two imaging sessions with two high-quality AIFs. (a) White-light image. (b) and (c) Fluorescence images at t=20  s of (b) the first and (c) the second imaging sessions, with the skin regions’ boundaries colored in red. (d) Individual AIFs (${\mathrm{AIF}}_{\mathrm{IND}}$) at each imaging session and the population-based AIF (${\mathrm{AIF}}_{\mathrm{POP}}$). (e)–(h) Boxplots of the average kinetic parameters (N=15) from (e) simple curve analysis ($I_{\max}$, TTP, IS) and (g) AATH modeling (BF, $K^{\mathrm{trans}}$, $k_{\mathrm{ep}}$) of ROIs within the targeted skin area (*paired t-test p<0.05) and corresponding CoV(%) of each parameter from (f) simple curve analysis (pre-AIF: before AIF correction) and (h) AATH modeling extracted based on pre-AIF, ${\mathrm{AIF}}_{\mathrm{IND}}$, or ${\mathrm{AIF}}_{\mathrm{POP}}$.

Assuming the blood perfusion in the normal intact skin area is consistent over two consecutive imaging sessions, Fig. 8 shows the comparison of the kinetic parameters in the skin areas [Fig. 8(a)] of an intraoperative patient case. This case involved two consecutive imaging sessions [Figs. 8(b) and 8(c)] with each of a corresponding high-quality AIF [Fig. 8(d)]. The kinetic parameters (N=15) were extracted either without AIF (pre-AIF) or based on ${\mathrm{AIF}}_{\mathrm{IND}}$ or ${\mathrm{AIF}}_{\mathrm{POP}}$. Red ROIs in Figs. 8(b) and 8(c) indicate targeted skin regions, with the low-intensity metal retractor and hyper-perfused skin regions near the incision area excluded.

In the simple curve analysis, as shown in Fig. 8(e), although the mean values of $I_{\max}$, TTP, and IS in the intact skin regions between two imaging sessions were very close to each other when corrected by ${\mathrm{AIF}}_{\mathrm{IND}}$, they were significantly different without AIF correction. In addition, when corrected by ${\mathrm{AIF}}_{\mathrm{POP}}$, besides TTP, $I_{\max}$ and IS were significantly different. Regarding the variability, the CoVs of $I_{\max}$ and IS corrected by ${\mathrm{AIF}}_{\mathrm{IND}}$ were lower than those based on ${\mathrm{AIF}}_{\mathrm{POP}}$ correction or without AIF correction. However, the CoV of TTP corrected by ${\mathrm{AIF}}_{\mathrm{IND}}$ was the highest among all [Fig. 8(f)].

Figure 8(g) demonstrated that the average BF in the intact skin regions remained consistent across two imaging sessions when analyzed using ${\mathrm{AIF}}_{\mathrm{IND}}$. However, significant differences were observed in BF when extracted by ${\mathrm{AIF}}_{\mathrm{POP}}$. Although variations in $K^{\mathrm{trans}}$ and $k_{\mathrm{ep}}$ across two imaging sessions were less influenced by which type of AIF was utilized, CoVs of these parameters extracted based on ${\mathrm{AIF}}_{\mathrm{IND}}$ were markedly lower than those based on ${\mathrm{AIF}}_{\mathrm{POP}}$ [Fig. 8(h)], suggesting that ${\mathrm{AIF}}_{\mathrm{IND}}$ can derive AATH parameters with fewer errors compared with ${\mathrm{AIF}}_{\mathrm{POP}}$.

### 3.4 Correlation Analysis Between Patient-Derived and Population-Average Parameters

For bone tissues, kinetic parameters extracted based on ${\mathrm{AIF}}_{\mathrm{IND}}$ and ${\mathrm{AIF}}_{\mathrm{POP}}$ within the bone regions were compared using 144 intraoperative imaging sessions of 110 patients. The evaluated kinetic parameters included BF, $K^{\mathrm{trans}}$, $k_{\mathrm{ep}}$, $I_{\max}$, TTP, and IS. In each of the 144 datasets, surgeons manually identified the exposed bone regions as ROIs, and the average values of each parameter were obtained.

**Fig. 9:**
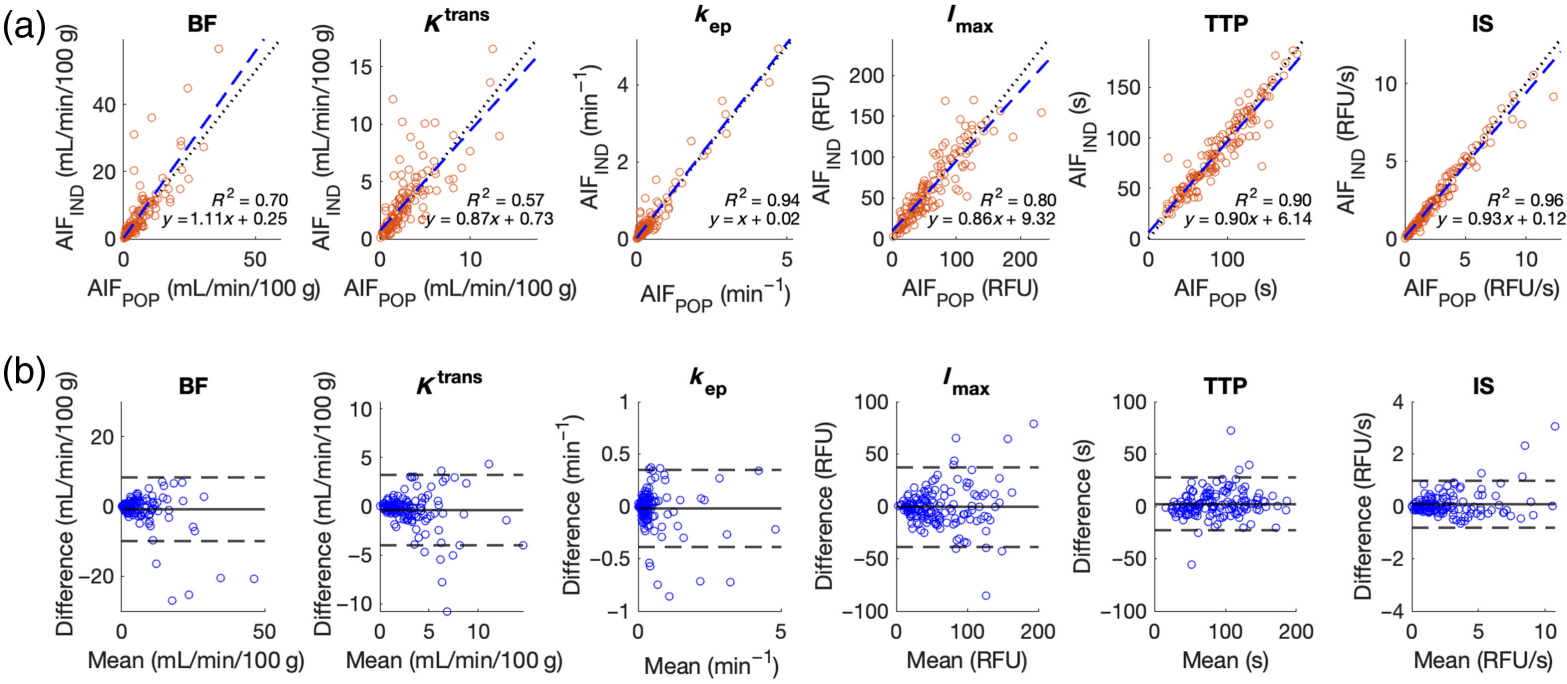
Comparison of ${\mathrm{AIF}}_{\mathrm{IND}}$ and ${\mathrm{AIF}}_{\mathrm{POP}}$ in perfusion-related kinetic parameters. Rows, from top to bottom, feature the regression plots (black dotted line: identity; blue dashed line: linear regression) and Bland–Altman plots (solid line: mean difference; dashed line: limits of agreement), respectively. Columns, from left to right, show the results from BF, $K^{\mathrm{trans}}$, $k_{\mathrm{ep}}$, $I_{\max}$, TTP, and IS.

In the regression plots in Fig. 9(a), the X and Y axes represented the average values based on either ${\mathrm{AIF}}_{\mathrm{POP}}$ or ${\mathrm{AIF}}_{\mathrm{IND}}$, respectively. The linear regression lines (blue dashed line) and annotated relevant metrics, such as slope, intercept, and $R^{2}$ on the lower-right corner of each figure, were also included. In the Bland–Altman plots in Fig. 9(b), the means of parameters extracted based on ${\mathrm{AIF}}_{\mathrm{IND}}$ and ${\mathrm{AIF}}_{\mathrm{POP}}$ served as X values, whereas the difference (${\mathrm{AIF}}_{\mathrm{POP}}-{\mathrm{AIF}}_{\mathrm{IND}}$) served as Y values. The mean difference (solid line) and the LOA (dashed line) are displayed in the figures. Additional detailed quantitative metrics are listed in Table 2.

**Table 2:** Comparison of each kinetic parameter extracted by ${\mathrm{AIF}}_{\mathrm{IND}}$ and ${\mathrm{AIF}}_{\mathrm{POP}}$ (N=144).

Parameter	Mean difference ± SD (unit)	p-Value	$R^{2}$
BF	−0.8 ± 4.7 (mL/min/100 g)	0.03	0.70
$K^{\mathrm{trans}}$	−0.4 ± 1.8 (mL/min/100 g)	0.02	0.57
$k_{\mathrm{ep}}$	−0.02 ± 0.2 ($\min^{-1}$)	0.15	0.94
$I_{\max}$	−0.7 ± 9.3 (RFU)	0.66	0.80
TTP	2.2 ± 2.9 (s)	0.04	0.90
IS	0.07 ± 0.5 (RFU/s)	0.07	0.96

As depicted in Fig. 9 and Table 2, relatively low correlations ($R^{2}=0.70$ and 0.57) and significant differences (paired t-test p<0.05) were found between the bone perfusion assessments of BF and $K^{\mathrm{trans}}$ based on ${\mathrm{AIF}}_{\mathrm{IND}}$ and ${\mathrm{AIF}}_{\mathrm{POP}}$, whereas $k_{\mathrm{ep}}$, $I_{\max}$, or IS exhibited favorable agreements with $R^{2}>0.8$. This suggests that ${\mathrm{AIF}}_{\mathrm{POP}}$ may be a viable replacement for ${\mathrm{AIF}}_{\mathrm{IND}}$ in estimating $k_{\mathrm{ep}}$, $I_{\max}$, and IS for perfusion assessments.

Table 3 presents the $R^{2}$ of the kinetic parameters in each imaging session of the amputation cohort, where each imaging session corresponds to a different level of perfusions, from the highest (baseline) to the lowest (soft tissue-stripping). With the decrease of perfusion (from baseline to tissue stripping), parameters including BF, $K^{\mathrm{trans}}$, $k_{\mathrm{ep}}$, and $I_{\max}$ exhibited substantially increased $R^{2}$ between the results extracted based on ${\mathrm{AIF}}_{\mathrm{IND}}$ and ${\mathrm{AIF}}_{\mathrm{POP}}$, suggesting a much closer agreement of these parameters for lower-perfused bone tissues. In contrast, both $R^{2}$ of TTP and IS were all larger than 0.9, regardless of the perfusion levels.

**Table 3:** Coefficient of determination ($R^{2}$) of each parameter extracted based on ${\mathrm{AIF}}_{\mathrm{IND}}$ and ${\mathrm{AIF}}_{\mathrm{POP}}$ in the amputation cohort at each bone damage level (N=44).

Parameter	$R^{2}$ of linear regression at each bone damage level		
	Baseline	Osteotomy	Tissue stripping
BF	0.29	0.36	0.78
$K^{\mathrm{trans}}$	<0.01	0.42	0.87
$k_{\mathrm{ep}}$	0.11	0.69	0.96
$I_{\max}$	0.69	0.80	0.92
TTP	0.92	0.98	0.97
IS	0.95	0.97	0.94

## 4 Discussion

This study successfully collected 144 high-quality AIFs using an oximeter-based PDD from 110 patients during open orthopedic procedures. These patient data were then utilized to validate the significance of AIF in tissue blood perfusion assessment using DCE-FI. To our knowledge, this is the first study to collect a large volume of AIFs based on oximeter-based PDD from a large representative orthopedic patient population during intraoperative ICG-based DCE-FI. The study further utilized these real AIFs to understand the role of the AIF in improving the accuracy of tissue vascular perfusion assessment through DCE-FI.

Figure 4 demonstrates large variations in the shapes and characteristics of AIFs across patients, whereas Fig. 5(a) shows that these variations in AIF cannot be fully explained by the differences in patient variables. A weakly positive correlation between $C_{\max}$ and weight (R=0.19) agrees with the fact that ICG dose was proportional to patient weight. However, all of the correlation coefficients from various regression analyses, including multivariate linear and non-linear regression, were below 0.4, indicating no significant relationship between AIF and patient variables. These results suggest that, currently, it is difficult to estimate AIF parameters solely based on patient information.

Due to the large variations in AIFs, ${\mathrm{AIF}}_{\mathrm{POP}}$ in Fig. 5(b) was calculated from a selected population (N=60). Averaging ${\mathrm{AIF}}_{\mathrm{POP}}$ from all patients (N=144) would smooth out the characteristic recirculation peak, potentially increasing errors in kinetic analysis. Deriving ${\mathrm{AIF}}_{\mathrm{POP}}$ across different cohorts is reasonable because the data collection process was the same, and the patient cohort did not differ significantly in key patient variables affecting ICG dose and AIF calculations, such as weight and hemoglobin (Table 1). Although differences in age and height were statistically significant and the imbalance of biological gender in the amputation cohort was seen in the daily clinical practice and related studies,^34^^,^^35^ they were not crucial factors affecting AIFs. Although it is possible to derive ${\mathrm{AIF}}_{\mathrm{POP}}$ for each cohort, for simplicity, only one ${\mathrm{AIF}}_{\mathrm{POP}}$ was derived to represent the entire patient cohort in this study.

In the simulation studies, errors arise from two main sources: signal noise in measured AIFs and computational errors in modeling. Signal noise in AIF primarily stems from inadequate estimation of signal amplitudes due to a series of filtering processes, which was mitigated through interpolations. Regarding computational errors, deconvolution employs a regularization parameter λ in Eq. (2) to ensure matrix invertibility, which may introduce errors in simple kinetic parameters ($I_{\max}$, TTP, and IS), because they are governed primarily by the high-frequency components of the tissue concentration curve. A small λ yields a noisy estimate, and a large λ may distort the output. Furthermore, the AATH model involves multivariate model fitting and is susceptible to optimization and convergence issues. To address these issues, we finetuned the value of λ within the range of 0.1 to $10^{-5}$. Within this range, $\lambda =10^{-3}$ was tested to adequately attenuate noise while maintaining data accuracy across the patient datasets. It achieved a noise reduction of 2.1 dB on average compared with $\lambda =10^{-5}$ and kept the root mean square error versus the maximum intensity within 6.4%, thus applied in the deconvolution process. A logarithmic L-curve between the residual and solution norms can be constructed to define the optimal λ for each case in the future study. To enhance the robustness of fitting in the AATH model, we also adopted multiple starting points of 3, 9, and 15  mL/min/100  g for BF and 5, 10, and 15 s for $t_{c}$, reducing modeling errors significantly. Starting points for $K^{\mathrm{trans}}$ and $k_{\mathrm{ep}}$ were fixed as these parameters were found to be stable regardless of starting points.

Both Figs. 6 and 7 indicate notably high error rates in $t_{c}$ in type I enhancement (low perfusion), making it less reliable for assessing low perfusion. This is because in type I, where fluorescence intensity steadily increases without clearance, the exponential decay (parenchymal phase) of the impulse residue function dominates the fitting, making the step function (vascular phase) more prone to errors. In contrast, $K^{\mathrm{trans}}$ and $k_{\mathrm{ep}}$ consistently demonstrate reliability across various enhancement types, as the fitting process is primarily influenced by the parenchymal phase.

In Fig. 8, we assessed the variability of kinetic parameters in the intact skin regions following two ICG injections in a specific case. Constrained by our clinical protocol, imaging FOV for amputation cases was predominantly occupied by bone regions with limited skin areas, and only a few cases with open fractures or infections underwent a second imaging session after further debridement. Furthermore, only a subset possessed multiple high-quality AIFs and large intact skin regions away from the borders of the incision area. In addition, as depicted in Fig. 8(d), the peak concentrations of AIFs in this case were notably different between the two injections, resulting in substantial changes in kinetic variables that mitigated the effects of computational errors.

The premise of this variability analysis, conducted across two imaging sessions of the same patient, was that perfusion in intact skin regions located at least 2 cm away from the incision area would exhibit minimal changes between sessions. This premise was supported by the insignificant differences in kinetic parameters extracted using ${\mathrm{AIF}}_{\mathrm{IND}}$ from both imaging sessions, as illustrated in Figs. 8(e) and 8(g), except for $k_{\mathrm{ep}}$. Notably, both $k_{\mathrm{ep}}$ obtained by ${\mathrm{AIF}}_{\mathrm{IND}}$ and ${\mathrm{AIF}}_{\mathrm{POP}}$ exhibited a significant increase (p<0.05) during the second imaging session following additional debridement. This suggests an elevation in the wash-out rate of ICG from the skin tissues to the blood vessels, despite the distance from the debridement site. This elevation could potentially be attributed to increased lymphatic drainage induced by the accumulated dosage of ICG during the second imaging.

We did not apply this analysis to bone tissues because bone perfusions inherently differ in cases involving multiple ICG injections. Due to clinical protocol, two consecutive imaging sessions without treatment for the bone were prohibited. Although bone perfusion varied significantly before and after orthopedic procedures such as osteotomy and soft tissue stripping, it is unfeasible to analyze the variability of kinetic parameters for bone tissues across multiple ICG injections.

In Figs. 8(f) and 8(h), the CoVs of kinetic parameters, including BF, $K^{\mathrm{trans}}$, $k_{\mathrm{ep}}$, $I_{\max}$, and IS, in skin regions following two ICG injections all decreased when extracted based on ${\mathrm{AIF}}_{\mathrm{IND}}$, compared with those based on ${\mathrm{AIF}}_{\mathrm{POP}}$ or no AIF correction. However, TTP was an exception, which exhibited the highest variability when extracted based on ${\mathrm{AIF}}_{\mathrm{IND}}$. This exception primarily stemmed from the proximity of $t_{\max}$ in ${\mathrm{AIF}}_{\mathrm{IND}}$ obtained from two ICG injections ($t_{\max}=10.9$ and 12.3 s, respectively). The variations in TTP corrected by ${\mathrm{AIF}}_{\mathrm{IND}}$ were overshadowed by the differences between ${\mathrm{AIF}}_{\mathrm{STD}}$ and ${\mathrm{AIF}}_{\mathrm{IND}}$ during deconvolution and re-convolution processes.

The analysis presented in Fig. 9 and Table 2 highlights the importance of extracting BF and $K^{\mathrm{trans}}$ from ${\mathrm{AIF}}_{\mathrm{IND}}$ rather than ${\mathrm{AIF}}_{\mathrm{POP}}$ for accurate perfusion assessment, whereas parameters such as $I_{\max}$ and IS can be computed using either method. BF, $K^{\mathrm{trans}}$, and TTP extracted from ${\mathrm{AIF}}_{\mathrm{IND}}$ were significantly different from those extracted from ${\mathrm{AIF}}_{\mathrm{POP}}$. Although BF and $K^{\mathrm{trans}}$ exhibited relatively low correlations ($R^{2}=0.70$ and 0.57, respectively) between the two types of AIF, TTP demonstrated a high correlation ($R^{2}=0.90$), suggesting disparities in TTP were more controlled and predictable. In contrast, $k_{\mathrm{ep}}$, $I_{\max}$, and IS showed close agreements between results calculated by ${\mathrm{AIF}}_{\mathrm{IND}}$ and ${\mathrm{AIF}}_{\mathrm{POP}}$, suggesting a possibility of using ${\mathrm{AIF}}_{\mathrm{POP}}$ for kinetic modeling when ${\mathrm{AIF}}_{\mathrm{IND}}$ is unavailable.

Table 3 illustrates that $R^{2}$ values of BF, $K^{\mathrm{trans}}$, $k_{\mathrm{ep}}$, and $I_{\max}$ based on ${\mathrm{AIF}}_{\mathrm{IND}}$ and ${\mathrm{AIF}}_{\mathrm{POP}}$ notably increase as the perfusion level decreases due to tissue damage. This may suggest that the errors by substituting ${\mathrm{AIF}}_{\mathrm{POP}}$ for ${\mathrm{AIF}}_{\mathrm{IND}}$ are smaller in poorly perfused tissues. The most significant increase in $R^{2}$ was observed in $K^{\mathrm{trans}}$. In baseline imaging with an average $K^{\mathrm{trans}}$ of 4.62  mL/min/100  g, there was a minimal correlation between $K^{\mathrm{trans}}$ based on ${\mathrm{AIF}}_{\mathrm{IND}}$ and ${\mathrm{AIF}}_{\mathrm{POP}}$ ($R^{2}<0.01$). However, following osteotomy and tissue stripping, the average $K^{\mathrm{trans}}$ dropped to 1.19  mL/min/100  g, whereas $R^{2}$ increased to 0.87, indicating a favorable agreement between $K^{\mathrm{trans}}$ obtained by two methods.

Although the significance of AIF has been demonstrated, it is important to acknowledge the limitations of this study. First, because the accuracy of AIFs collected by oximeter-based PDD has been validated with AIFs based on blood sampling at multiple time points in animal and human studies,^22^^,^^36^ we excluded the validation of the accuracy of the collected AIFs due to restrictions in our clinical protocol that prevented multiple blood sample collections during surgeries. Although the validation of blood flow could not be carried out, the accuracy of blood flow estimations can be improved using AIFs collected by oximeter-based PDD, which has been validated in multiple animal and human studies.^37^^,^^38^ Future studies using fluorescent microspheres and *in vitro* microtubing flow phantoms can be conducted to quantitatively validate the blood perfusion obtained through near-infrared imaging combined with pulse oximetry.^39^^,^^40^ Blood flow can be estimated using compact and low-cost handheld laser speckle imaging on relatively clean and flat tissue surfaces without requiring an injection of contrast agent, based on speckle pattern analysis.^41^ However, as discussed in our previous paper,^29^ this imaging modality involves laser speckles that can be significantly influenced by surface blood and the 3D structure of bone surfaces, making it difficult to adapt for intraoperative bone imaging.

Second, AIF in each case is applied as a global curve regardless of the measurement location, although its shape can be distorted by dispersion during transit from the injection site to the target tissue.^6^ Due to the patient imaging protocol, AIF measurement has been limited to the fingers of the hand opposite to the ICG injection arm. However, the specific finger was randomly chosen for each case, as the differences between the fingers of the same hand did not significantly affect AIF.

Third, ${\mathrm{AIF}}_{\mathrm{POP}}$ in this study was averaged from the selected high-quality AIF datasets, neglecting case-specific differences, which may explain its limited effectiveness in reducing the variability of kinetic parameters compared with ${\mathrm{AIF}}_{\mathrm{IND}}$. However, individual variations could be incorporated into generalized ${\mathrm{AIF}}_{\mathrm{POP}}$, as several studies have attempted to modify ${\mathrm{AIF}}_{\mathrm{POP}}$ based on patient information, such as body weight, cardiac output, and blood volume.^42^^–^^44^ Future work will investigate whether adjusted or subtype ${\mathrm{AIF}}_{\mathrm{POP}}$ could exhibit better agreements with ${\mathrm{AIF}}_{\mathrm{IND}}$, potentially aiding in the correction of noisy or corrupted data.

Finally, the clinical relevance of the differences between ${\mathrm{AIF}}_{\mathrm{IND}}$ and ${\mathrm{AIF}}_{\mathrm{POP}}$, as depicted in the Bland–Altman plots of Fig. 9(b), has not yet been fully understood. This is primarily due to the significant heteroscedasticity in the differences, which could not be resolved using normalization or logarithmic transformation for parameters such as BF, $K^{\mathrm{trans}}$, $k_{\mathrm{ep}}$, $I_{\max}$, and IS. Consequently, V-shaped 95% confidence limits were constructed,^33^ illustrating a substantial reduction in kinetic differences estimated based on ${\mathrm{AIF}}_{\mathrm{IND}}$ and ${\mathrm{AIF}}_{\mathrm{POP}}$ in cases with lower perfusion levels, consistent with the findings presented in Table 3. Further research is necessary to establish thresholds in the kinetic parameters based on clinical outcomes before determining the clinical significance of the differences in each kinetic parameter between ${\mathrm{AIF}}_{\mathrm{IND}}$ and ${\mathrm{AIF}}_{\mathrm{POP}}$.

## 5 Conclusion

The results from this study indicated that patient-specific ${\mathrm{AIF}}_{\mathrm{IND}}$ collected from PDD were able to reduce the variability in kinetic parameters and improve the accuracy of perfusion assessment when using DCE-FI. ${\mathrm{AIF}}_{\mathrm{IND}}$ can provide the most accurate perfusion assessment compared with that without AIF or based on ${\mathrm{AIF}}_{\mathrm{POP}}$ correction. ${\mathrm{AIF}}_{\mathrm{POP}}$ averaged from high-quality ${\mathrm{AIF}}_{\mathrm{IND}}$ has the potential to replace ${\mathrm{AIF}}_{\mathrm{IND}}$ for estimating $k_{\mathrm{ep}}$, $I_{\max}$, and IS with relatively lower differences compared with those for estimating blood flow, $K^{\mathrm{trans}}$, and TTP. More research is needed to evaluate the clinical significance of the differences in kinetic parameters between ${\mathrm{AIF}}_{\mathrm{IND}}$ and ${\mathrm{AIF}}_{\mathrm{POP}}$.

## Data Availability

Data underlying the results presented in this paper are not publicly available at this time but may be obtained from the authors upon reasonable request.
